# Natural Resource Management Schemes as Entry Points for Integrated Landscape Approaches: Evidence from Ghana and Burkina Faso

**DOI:** 10.1007/s00267-017-0866-8

**Published:** 2017-04-20

**Authors:** Samson Foli, Mirjam A. F. Ros-Tonen, James Reed, Terry Sunderland

**Affiliations:** 1 Department of Geography, Planning and International Development Studies and Centre for Sustainable Development Studies, University of Amsterdam, P.O. Box 15629, Amsterdam, 1001 NC The Netherlands; 2 Center for International Forestry Research (CIFOR), Jalan CIFOR, Situ Gede,, Bogor, West Java 16115 Indonesia

**Keywords:** Natural resource management, Landscape approach, CREMA, Modified taungya system, Chantier d’Aménagement Forestier

## Abstract

In recognition of the failures of sectoral approaches to overcome global challenges of biodiversity loss, climate change, food insecurity and poverty, scientific discourse on biodiversity conservation and sustainable development is shifting towards integrated landscape governance arrangements. Current landscape initiatives however very much depend on external actors and funding, raising the question of whether, and how, and under what conditions, locally embedded resource management schemes can serve as entry points for the implementation of integrated landscape approaches. This paper assesses the entry point potential for three established natural resource management schemes in West Africa that target landscape degradation with involvement of local communities: the Chantier d’Aménagement Forestier scheme encompassing forest management sites across Burkina Faso and the Modified Taungya System and community wildlife resource management initiatives in Ghana. Based on a review of the current literature, we analyze the extent to which design principles that define a landscape approach apply to these schemes. We found that the CREMA meets most of the desired criteria, but that its scale may be too limited to guarantee effective landscape governance, hence requiring upscaling. Conversely, the other two initiatives are strongly lacking in their design principles on fundamental components regarding integrated approaches, continual learning, and capacity building. Monitoring and evaluation bodies and participatory learning and negotiation platforms could enhance the schemes’ alignment with integrated landscape approaches.

## Introduction

In sub-Saharan Africa, close to 600 million people are dependent on forests for food and/or income, and many of those are food insecure (Ickowitz et al. 2014; Koffi et al. 2016). Facing insecurities related to forest decline, loss of biodiversity and erosion of ecosystem services, many are also vulnerable to the impacts of climate change (Bele et al. 2015; Mantyka-Pringle et al. 2015). Recent environmental discourse calls for the integrated governance of natural resources and integrating conservation efforts with opportunities for rural economic development and increased resilience to climatic vicissitudes (Sachs et al. 2009; Scherr et al. 2012; Minang et al. 2014; Sunderland et al. 2015).

An integrated landscape approach is a framework that attempts to reconcile social, environmental, and economic development with biodiversity conservation and climate change mitigation (Kozar et al. 2014). Although seen as a departure from sectoral approaches to land management, rather than seeking elusive win–win outcomes, landscape approaches recognize that trade-offs between socio-economic and ecological objectives can—and will—occur. Several authors have developed principles and tools that can guide research and decision-making on multifunctional landscapes (Sayer et al. 2013; Ros-Tonen et al. 2014; Reed et al. 2015; Chia and Sufo 2016), yet it remains unclear how these principles can be fully operationalized in specific contexts (Reed et al. 2017).

Furthermore, a natural resource governance approach that recognizes multiple and often competing land uses and manages the trade-offs between them is complex, time- and cost-intensive (Reed et al. 2015, 2016) and long-term funding poses a challenge (Hart et al. 2014). Identifying locally embedded natural resource management (NRM) initiatives that can provide entry points for integrated landscape approaches offers potential to overcome some of these implementation challenges. Reviewing one NRM scheme in Burkina Faso and two in Ghana, this paper aims to highlight whether, and how, differing NRM initiatives that integrate landscape and livelihood objectives of local populations can provide such entry points. Hence this paper asks, to what extent do three locally embedded resource management initiatives in Burkina Faso and Ghana align with the fundamental design principles of integrated landscape approaches and what does this mean for their potential to function as entry points for implementing integrated landscape approaches?

The next section briefly describes the relevant NRM schemes and their importance within their respective local landscape contexts. The methodology section justifies the selection of the cases and details the literature review and analytical framework. The results section analyzes the characteristics of the three schemes along the major design principles for integrated landscape approaches. This serves as a basis for the discussion in which we assess the potential of the schemes to act as entry points for integrated landscape approaches. The conclusion explains the implications of the findings.

### Forested Landscapes in Burkina Faso and Ghana: Status and Threats

Burkina Faso had 5.4 million hectares of forests as of 2015; 95.5% naturally regenerated and 4.5% planted forests (FAO 2015, p. 34). Most of Burkina’s forests have less than 40% canopy cover (Paré et al. 2008; Pouliot and Treue 2013) and deforestation ranged between 0.77 and 1.2% per annum^1^ from 1990 to 2010 (Ouedraogo 2006; Westholm and Kokko 2011; FAO 2015). Burkina’s dry forests constitute a fragmented arable landscape dominated by agroforestry parklands interspersed with deciduous shrub, woody savannah characterized by economically valuable tree species such as shea (*Vitellaria paradoxa*) and parkia (*Parkia biglobosa*) (Boffa 1999; Arbonnier 2004; Ky-Dembele et al. 2007). These multifunctional land-use mosaics supply food, fodder, and fuelwood to both rural and urban populations (Ouédraogo 2009; Foli and Rabdo 2016). Trees and non-timber forest products (NTFPs) serve as important food safety nets for the most vulnerable farmers during lean seasons (Koffi et al. 2016). The forested landscape faces pressures of climate-change induced migration flows from the north (Ouedraogo et al. 2010); uncontrolled felling of trees to supply the commercial fuelwood trade; and high fuelwood demand to meet the energy needs of major urban areas (Kabore and Ouedraogo 2000; Ouédraogo 2009).

Ghana’s forest cover of 41% (9.3 million hectares) encompasses primary forest (4.2%), naturally regenerated forest (92.3%) and planted forests (3.5%) (FAO 2015, p. 229). Tree cover decreases from the high forest zone in the south-western third of the country toward the transitional and savannah zones of the north (Derkyi 2012). Forest reserves cover 16.2% of Ghana’s total land area, 80% of which is production forest designated for timber exploitation, and 20% as protection reserves for conservation purposes (MLNR 2012). Major threats to the forests are illegal farming, chainsaw logging and—in some areas—artisanal and small-scale mining (Derkyi 2012; Hilson 2012). Moreover, national policies have been instrumental to the introduction and intensification of cocoa and, more recently, oil palm (Ros-Tonen et al. 2015), both of which are considered major threats to the remaining forests (Benhin and Barbier 2004; Appiah et al. 2009).

### NRM Schemes in Ghana and Burkina Faso Targeting Landscapes and Livelihoods: A Brief Overview

To address the degradation of forested landscapes, Ghana and Burkina Faso have experimented with several co-management and community-based NRM initiatives since the 1930s and 1970s, respectively (Agyeman 2006; Murphree 2008; Sawadogo and Tiveau 2011; Asare et al. 2013). These initiatives were established in forest zones with high population pressure where smallholders depend on forests for natural resources.

In Burkina Faso, forest resource management involving rural communities was piloted in 1985 in the Nazinon forest reserve of Sissili Province in the Center-West Region with international donor support and joint implementation by the United Nations Development Program (UNDP) and the Food and Agricultural Organization of the United Nations (project UNDP/FAO/BKF/85/011). Offices of the Ministry of Forest and Environment and Sustainable Development (MEDD) were established in the communities and out-posted technicians to provide training on improved methods of tree felling and regenerative coppicing and informing forest management cooperatives (GGF, Groupement de Gestion Forestière). The Chantier d’Aménagement Forestier (CAF) scheme regulates fuelwood harvesting by issuing permits to the forest management groups. Felling permits detail the quantity and type of wood species to harvest from the forest reserve. The revenues of fuelwood trade are distributed on a ratio of 9.1, 13.6, 27.3, and 50%, respectively for the forest tax (harvesting permit), contribution to the village development fund, the forest management fund, and the forest management group members (Ouédraogo 2007; Coulibaly-Lingani et al. 2011). The forest tax supports and staffs CAF offices, whereas the forest management fund supports communities to carry out annual tree planting and monitor the biodiversity of forests (Ouédraogo 2007). Approximately 350,000 ha of forests are currently managed by autonomous unions and cooperative groups under the CAF scheme (Paré et al. 2008; Ouedraogo et al. 2009). In 2012, there were 12 forest management sites (CAFs), involving 473 community forest management groups with 12,000 members officially recognized by the MEDD (2012).

The modified taungya system (MTS) in Ghana is a reforestation scheme launched in 2002 to replace the original taungya introduced in the 1930s in response to rapid forest loss within reserves due to the high demand of forest resources by then colonial authorities (Kalame et al. 2011). The scheme allowed the intercropping of food crops in the early part of the plantation establishment cycle. Approximately 75,000 ha of taungya plantations were established in the 1970s (Agyeman 2006), but the scheme was suspended in 1984 due to a lack of support from farmers who lacked long-term incentives as they were not entitled to shares in timber revenues (Agyeman et al. 2003; Blay et al. 2008; Kalame et al. 2011). The MTS is a co-management arrangement between the Ghana Forestry Commission and local communities and differs from the previous system in that (a) farmers are now entitled to 40% of the timber proceeds to compensate for their efforts in tree planting, maintenance and protection, and (b) institutions were established to provide farmers a voice in management decisions (Ros-Tonen et al. 2013, 2014; Acheampong et al. 2016). A total of 94,115 ha was planted under the MTS between 2002 and 2009 (FC 2015).

CREMAs were launched in Ghana by the Wildlife Division of Ghana’s Forestry Commission^2^ in the early 2000s to curtail destruction of wildlife habitats in the country’s off-reserve forest areas (Murphree 2008; Asare et al. 2013). The scheme was framed as decentralized and participatory NRM for wildlife conservation and livelihood diversification, while acting as a habitat buffer at the fringes of protected areas and wildlife reserves (Baruah 2015). The guiding principles represent a paradigm shift in Ghana’s wildlife policy by giving the local population an increased say over natural landscapes and granting official access to economic benefits from natural resources, thereby recognizing the roles of traditional authorities and women (Asare et al. 2013; WD 2000). There were 26 active CREMAs in 2010 (Asare et al. 2013) and currently over 30 CREMAs in various stages of establishment (Baruah 2015). In total, 313,934 hectares of land are incorporated within CREMA sites, involving 215 communities and satellite communities (Asare et al. 2013).

Further details on the scope of the schemes and actors involved are presented in Table 1.

**Table 1:** Characteristics of the reviewed natural resource management initiatives

Scheme	Summary	Actors (individuals and associations) involved	Management concept	Natural resources managed	Scope of land uses concerned	Key references
Chantier d’Aménagement Forestier (CAF), Burkina Faso	Decentralized and participatory forest management program to stem deforestation resulting from fuelwood harvesting and land degradation throughout Burkina Faso	• Local offices of Ministry of environment and sustainable development (MEDD): training in sustainable wood harvesting and forming cooperatives; supporting the audit committee	Co-management between MEDD’s provincial offices, CAF directorate and cooperatives/Forest management units	Delimitation of forest plots with controlled annual harvesting rotations. Protection and replanting of threatened tree species	Production forests each sub-divided for 20–25 year rotational harvesting	Sawadogo (2007), Ouédraogo (2009), Sawadogo and Tiveau (2011), Coulibaly-Lingani et al. (2011), Foli and Rabdo (2016), Arevalo (2016)
		• CAF Management Board: administration of forest sites and enforcement of statutory laws and voluntary regulations				
		• CAF Audit Committee: audit efficacy of the quota system on wood volumes harvested, sales, taxes and revenues (financial arm) and monitoring wood extraction and regeneration activities (technical arm)				
		• CAF technical unit: technical support to the Forest Management Groups’ activities				
		• Forest management group: cooperative of woodcutters responsible for controlled wood harvesting and users of non-timber forest products				
		• Union of cooperatives that utilize the same forest sites				
		• External partners: funding CAF activities, technical support and capacity building of CAF technical units				
Modified Taungya System (MTS), Ghana	Forest restoration program in degraded forests with farmers engaged in tree planting and maintenance and benefitting from intercropping of food crops and a share in timber revenues	• Forestry Commission (FC): supplying seedlings, training and extension; marketing the plantation products; overall supervision; financial management	Co-management with responsibilities and benefits shared between the state (Forestry Commission), small-scale farmers and local communities	Degraded forest reserves with a focus on restoration	Progressive transformation of degraded forest reserves via early agroforestry systems (intercropping with food crops) to plantation forests	Agyeman et al. (2003), Blay et al. (2008), Kalame (2009), Kalame et al. (2011), Ros-Tonen et al. (2013, 2014), Acheampong et al. (2016)
		• Forest services division: the FC’s district representation				
		• Resource Management Support Center (technical wing of the FC): supports the implementation of the MTS				
		• MTS farmers: tree planting and maintenance				
		• Administrator of Stool lands and traditional authorities: providing secure and uninterrupted access to land in the degraded forest area				
		• Local community: preventing wildfires and timber theft				
		• (Optional:) Timber company: timber marketing				
		• (Optional:) NGO: developing alternative livelihood activities				
Community resource management areas (CREMAs), Ghana	CREMAs are part of policy reforms aiming to reconcile wildlife conservation, rural development and livelihoods in off-reserve areas. Spearheaded by the Wildlife Division with on-going devolution to local communities	• FC (National): enforcement of forest and wildlife laws	Adaptive co-management	Wildlife and habitat conservation in off-reserve areas and in zones fringing protected areas	Integrated local level wildlife conservation within productive land uses such as agriculture, hunting, and timber and non-timber forest product extraction	Murphree (2008), Wildlife Division (2000), Nyame et al. (2012), Robinson et al. (2012), Agyare (2013), Asare et al. (2013), Robinson and Sasu (2013), Agyare et al. (2015a, b), Baruah (2015)
		• Wildlife Division of the FC: catalyst of the CREMA, advice and monitoring				
		• Forest Services Division: district representation of the FC				
		• Ministry of Lands and Natural Resources (MLNR): registers the CREMA and is responsible for sustainable natural resource management in off-reserve areas				
		• NGOs (e.g., Care International, IUCN, ARocha): often initiate the CREMA process and mobilize communities				
		• District Assemblies: development services; formalizing CREMA rules and regulations; issuing wildlife trading permits				
		• Administrator of Stool lands: providing secure access to land				
		• Traditional authorities: involved in CREMA administration and conflict management				
		• CREMA communities/cluster of communities: engage in CREMA activities				
		• Elected Community Resource Management Committee (CRMC) (5–13 men and women): decision-making body for CREMA implementation				
		• Elected CREMA Executive Committee: steering and overseeing daily operations and decision-making				
		• (Optional:) Protected Area Management Advisory Board (PAMAB): assists in the management of protected areas				
		• (Optional:) Ministry of Food and Agriculture (MOFA): agricultural training and extension				
		• (Optional:) Timber company: responsible for the marketing of timber if a CREMA embarks on logging				

## Materials and Methods

### Selection of Cases

The three schemes described in the previous section were selected because they were, (1) designed to address landscape degradation while recognizing the long-standing natural resource demands and livelihood objectives of inhabitants, and (2) are the principal NRM schemes in the two countries involving local communities and other stakeholders.

### Literature Review

The literature referred to in this study (national policy documents, project implementation and assessment reports, and peer-reviewed literature) was gathered in four steps. First, gathering peer-reviewed sources involved a search through the Web of Science database, using the names of schemes and their acronyms. Second, additional ‘gray literature’ was sought using the same keywords in Google Scholar. Publications that focused on the performance and governance aspects of the schemes were selected for further review. Third, unpublished documents were found through the researchers’ previous project work in both countries. For Burkina Faso, relevant policy documents and published and unpublished literature was acquired during scoping visits to identify project sites and research partners for the “Sustainable Use of Tropical Forest Biodiversity” project. Similar data on the schemes in Ghana was gathered through long-established contacts with the Wildlife Division and Research Management Support Center—two divisions of the Ghana Forestry Commission responsible for the respective schemes. Fourth, bibliographies of source documents were assessed to identify additional articles, reports, and outputs. Key literature on integrated landscape approaches was known to the authors based on a prior review (Reed et al. 2016).

### Analytical Framework

The analytical framework used for this study builds on the authors’ past work on design principles for integrated landscape approaches (Sayer et al. 2013; Ros-Tonen et al. 2014). The key principles used to analyze the extent to which the three schemes align with landscape approaches and the scoring used are presented in Tables 2 and 3.

**Table 2:** Landscape approach principles used for the analysis of the three schemes

Principle^a^	Elaboration	Reference
Integrated approach	Integrated approaches recognize the need to reconcile multiple land uses and negotiate trade-offs between land uses, notably those between conservation and economic development objectives of people and/or communities living in around a natural resource base	Folke et al. (2005), Harvey et al. (2008), DeFries and Rosenzweig (2010), Sayer et al. (2013), Reed et al. (2016)
Adaptive management and continual learning	This principle acknowledges the physical and socio-economic dynamics in landscapes and the need to instill continual learning, willingness to adapt management practices as well as underlying assumptions, norms and principles, thereby accepting a diversity of solutions, actors and institutions	Dietz et al. (2003), Berkes (2009), Armitage (2005), Gupta et al. (2010), Koffi et al. (2016)
Polycentric governance	Polycentric governance in the context of landscape approaches recognizes multiple and multilevel centers of decision making, including statutory, customary and hybrid ones	Phelps et al. (2010), Nagendra and Ostrom (2012), Pahl-Wostl (2015)
Multi-stakeholder negotiation	This concerns the need to involve nearby and distant stakeholders in landscape governance and the understanding that land uses, common goals and trade-offs need to be continuously negotiated	Lebel et al. (2006), Balint et al. (2011), Sayer et al. (2013), Kozar et al. (2014)
Capacity building	In order to enhance the equity of actors in processes of self-organization, innovation, monitoring and evaluations of resource governance, actors need to possess a certain level of know-how and experience on relevant issues. Capacity building of involved actors, especially local representatives, grassroots collectives and implementers of resource governance activities is a required component to create a level playing field during negotiation processes	Fakuda-Parr and Lopes (2013), Eade (2007), Virji et al. (2012), Sayer et al. (2013), Clark et al. (2016)

^a^ Based on Ros-Tonen et al. (2014) synthesized from the ten principles of landscape approaches from Sayer et al. (2013)

**Table 3:** Details used for scoring design principles of a landscape approach

Score	Very weak 1	Weak 2	Moderate 3	Strong 4	Very strong 5
Principle					
Integrated approach	No or hardly any integration of conservation and development aims	Multifunctionality embraced, but management focus on one	Multifunctionality embraced; trade-offs acknowledged, but decided in favor of dominant use	Multifunctionality embraced; trade-offs acknowledged and negotiated, but not beyond the scheme	Multifunctionality embraced; trade-offs acknowledged and negotiated with broader set of stakeholders
Adaptive management and continual learning	Need to adapt to physical and socio-economic dynamics not/hardly acknowledged	Single-loop learning^a^: willingness to improve daily routines	Single and double-loop learning: willingness to reframe assumptions	Single, double and triple-loop learning: willingness to challenge underlying norms and values and accept a diversity of solutions, actors and institutions^b^	Triple-loop learning based on participatory monitoring and evaluation; room for autonomous change^b^
Polycentric governance^c^	Single center of decision-making	Co-governance with joint responsibility for setting the rules	Multi-level governance and decision-making with openness to include non-state actors (civil society, private sector)	Networked governance: mechanisms in place for horizontal and vertical interaction between operationally autonomous players	Hybrid governance: interactive decision-making involving actors at different levels and scales (horizontal and vertical)
Multi-stakeholder negotiation^d^	One actor dominates in setting goals, targets and change logic; stakeholders informed	Mechanisms in place to negotiate land use and production targets, but hardly used	Consensus about objectives, options, and targets, but no negotiation of trade-offs	Shared vision on land uses and change logic; limited space to negotiate trade-offs	Objectives and change logic negotiated based on FPIC^e^; stakeholders negotiate about trade-offs considered as acceptable
Capacity building (CB)	Knowledge dissemination	CB focuses on transferring skills that dominant actor considers as “desirable”	CB aligns with local knowledge and needs and targets collaborative capacity	CB enhances negotiation skills and inclusive decision-making^f^	CB enhances adaptive capacity and acts as “catalyst of change”^g^, and empowerment

^a^ For the distinction between single, double and triple loop learning see Armitage et al. (2008) and Pahl-Wostl (2009)

^b^ Based on Gupta et al. (2010)

^c^ Operationalization based on Paletto et al. (2011) and Nagendra and Ostrom (2012)

^d^ Operationalization inspired by Sayer et al. (2013)

^e^ Free, prior, and informed consent

^f^ Foster-Fishman et al. (2001)

^g^ Virji et al. (2012)

## Results

This section discusses the features of the three NRM schemes along the five design principles highlighted in Table 2.

### The CAF

#### Integrated approach

CAF forests are located within multifunctional parkland agroforestry landscapes that highlight historical integration of forests and food production (Gautier et al. 2015; Westholm 2016). This stems partly from frequent climate vulnerabilities and resource degradation (soil and land degradation) of the region and the search for food safety nets (Koffi et al. 2016). Productive land uses, principally cereal cropping and agro-pastoralism, are often combined with the harvest, processing and sale of wood and NTFPs, which in turn contribute to food security (Boffa 1999; Koffi et al. 2016). Historical importance of forests is emphasized in customary laws that commonly protect important tree species from felling throughout the country (Coulibaly-Lingani et al. 2011; Ouédraogo 2009). The CAF itself does not explicitly pursue an integrated approach beyond the demarcated production forests set aside for fuelwood extraction.

#### Adaptive management and continual learning

CAF was implemented as a co-management scheme of forest between locally chosen representatives, a technical arm and statutory bodies—from the Ministry of Environment down to regional, provincial and the CAF technical body at the district level (Sawadogo 2007; Arevalo 2016). Learning, reciprocity and exchange of information were minimal prior to the scheme becoming autonomous in the early 2000s. In the last decade, learning within the CAF has been implemented through research and donor projects targeting forest technicians who support CAF activities and user groups. The latter are predominantly woodcutters who are targeted with updated knowledge regarding felling techniques and directives from the MEDD and women groups supported in setting up tree nurseries and vegetable gardens (Etongo et al. 2015).

#### Polycentric governance

Organizational format of CAF schemes involves a variety of actors (Table 1). Forest management groups (GGF) are cooperatives at community level made up of woodcutters autonomously exploiting fuelwood from the demarcated parcels (Coulibaly-Lingani et al. 2011). GGFs utilizing a common CAF parcel (Unité d’Aménagement Forestier) form Unions of Forest Management Groups (UGGFs or Union des Groupements de Gestion Forestière) (Kabore and Ouedraogo 2000). General administration of the CAF is responsibility of the CAF Management Board (Table 1), which ensures compliance with voluntary CAF regulations, laws of the Forestry Service, and national rules governing cooperatives. The Board comprises presidents of individual forest management groups and unions, appointed by the collective bureaus of UGGFs (Kabore and Ouedraogo 2000). The Board, forest management groups and unions receive support from the CAF Audit Committee in administrating fuelwood quotas, sales, taxes and revenues, and from the Technical Unit for technical matters regarding tree felling, rotation, tree cutting methods, and regeneration (Agbor and Tanyi 2015; Foli and Rabdo 2016). Despite the high complexity, CAF governance maintains a hierarchical structure and therefore does not represent an example of polycentric governance.

#### Multi-stakeholder negotiation

CAF groups at district level meet periodically in community-level groups (GGFs) and cooperative unions (UGGFs) and both meet with the Audit Committee annually. Technicians frequently meet with village-level GGFs as part of a biodiversity monitoring routine. Provincial and regional MEDD offices also meet annually to evaluate performance of conservation priorities and to renew objectives. UGGFs are invited to attend, but they usually delegate attendance to the CAF technical director who has the capacity to engage in such meetings. Whether the technical director attends depends on availability of resources. There are no dedicated platforms where all actors of the scheme can collectively negotiate conservation objectives and challenges. Local CAF organizations often request intermediaries— from either CAF Technical Units, NGOs, or research institutions—to negotiate conservation goals to the regional MEDD on their behalf (Westholm 2016). Hence, negotiation structures are in place, but resources to enable local actors to access discussions at regional and national level are frequently lacking, inhibiting their effective involvement in decision-making concerning forest management.

#### Capacity building

Per CAF regulations, technicians receive trainings and refresher conservation education annually, but from record, technicians in the CAF may go for 3 years without receiving new training. The CAF is dependent on external NGO projects for capacity building or trainings on new forest management techniques. There is no explicit focus on capacity building within the CAF beyond the formation of cooperatives and cooperative unions. This limits the capacity of woodcutters and their representatives to negotiate trade-offs of competing land use between them.

### The MTS

#### Integrated approach

The MTS exhibits characteristics of an integrated approach by combining timber production, intercropping of food crops, carbon sequestration and climate change adaptation (Kalame et al. 2011; Ros-Tonen et al. 2014; Lasco et al. 2014). Income is derived from the sale of food crops, seedling production and re-investment of revenues in petty trade, resulting in increased food security, improved housing and uptake in school attendance among children (Derkyi 2012; Insaidoo et al. 2013; Ros-Tonen et al. 2014). These benefits are however transitory as inter-planting food crops is possible until canopy closure (3 years on average), while long-term timber benefits are subject to risks of theft and wildfire (Ros-Tonen et al. 2014; Acheampong et al. 2016). Measurements of the carbon potential of MTS have been disappointing (Yeboah et al. 2014), but its contribution to climate change adaptation may be promising (Lasco et al. 2014). However, the scheme is still primarily designed to produce timber and, despite an ongoing pilot to introduce shade-tolerant non-timber products^3^, as yet, there is no policy to forge an integrated approach.

#### Adaptive management and continual learning

Learning in the MTS usually refers to training in tree planting and seedling production, mainly organized by the Forest Services Division in the form of field visits (Insaidoo et al. 2013; Ros-Tonen et al. 2013). Learning has seldom been the result of institutionalized reflection and evaluation, hence does not reflect adaptive management. An exception was the relaunch of the MTS in 2002 that, following stakeholder consultations, drew on the mistakes and disappointments of the colonial taungya system. Current discussions about introducing shade-tolerant species as a way to enhance the livelihood benefits of the scheme hint at a collaborative learning process. Overall, however, continual learning has been limited as the MTS was not designed as an adaptive management system (Ros-Tonen et al. 2014).

#### Polycentric governance

The MTS was intended to give greater voice to farmers through the Land Allocation and Taungya Management Committee (‘the Taungya Committee’) (Acheampong et al. 2016). This committee is responsible for (i) allocating degraded forest reserve land to MTS farmers, (ii) monitoring performance and ensuring compliance of all parties with the contract, and (iii) instituting sanctions and settling disputes (Agyeman et al. 2003). During years of financial support from the African Development Bank (AfDB) to the Community Forest Management Project (between 2002–2010) (which included the MTS), associations or task forces emerged that undertook joint social activities such as alternative livelihood projects; funeral donations to bereaved members; communal labor in pegging and planting; and monitoring theft and fire risks (Ros-Tonen et al. 2013; Osei-Tutu et al. 2015; Acheampong et al. 2016). Occasionally, such associations also designed additional local by-laws to guide the implementation of the MTS (Insaidoo et al. 2013; Ros-Tonen et al. 2013). These rules, however, focused on compliance with, and effective implementation of rules established by the Forestry Commission. In practice, the latter sets the rules regarding tree and food-crop choice and specific farming practices that farmers should use (Derkyi 2012; Ros-Tonen et al. 2014). The MTS Agreement defined by the Forestry Commission stipulates the rules regarding responsibilities, inputs and benefit-sharing (Agyeman et al. 2003; FC 2006). Although the governance structure of MTS encourages links across jurisdictional scales (local authorities, stools, and national government), institutional scales (the constitution, operating rules, by-laws) and spatial scales (from local to national), hybridity and polycentric decision-making within the scheme is hardly recognized. Despite the space for the taungya committees to design locally-specific by-laws, hierarchical governance prevails (Derkyi 2012; Ros-Tonen et al. 2014; Acheampong et al. 2016). Furthermore, polycentric governance is hindered by weak local institutions being poorly connected to external organizations (Akamani et al. 2015).

#### Multi-stakeholder negotiation

After the failure of the colonial taungya system, the World Bank and the Food and Agricultural Organization of the United Nations (FAO) financed a stakeholder consultation in 2001–2002 during which farmers, communities, land owners and NGOs were consulted on the design of the MTS (Ros-Tonen et al. 2014). This resulted in a redesign of the scheme with more democratic governance and a benefit-sharing arrangement that entitles farmers to 40% of timber proceeds (Marfo 2009). Subsequently, the MTS offers a multi-stakeholder design accommodating the four actor groups outlined in Table 1. Broader partnerships with public actors, donors, NGOS and, in some cases, the private sector were formed where the MTS was implemented under the AfDB-financed Community Forest Management Project (Marfo et al. 2012; Ros-Tonen et al. 2014). However, such partnerships remain dependent on donor funding and space to negotiate the conditions of the scheme or propose a different course remains limited.

#### Capacity building

Capacity building in the MTS encompasses training in seedling production and tree planting, with attention to ‘alternative livelihoods’ (e.g., small livestock rearing) during the years of AfDB support to the community forest management plan. This type of training focuses on transferring skills rather than enhancing farmers’ negotiation capacity and empowerment (Insaidoo et al. 2013; Ros-Tonen et al. 2013, 2014).

### CREMA

#### Integrated approach

An integrated land-use approach is inherent in CREMA governance: CREMAs attempt to reconcile competing land uses in order to manage trade-offs between human activities and wildlife conservation. Eco-tourism and the exploitation of NTFPs, including wildlife, are popular land uses in this respect, for generating income while (allegedly) preventing the conversion of wildlife habitat into farmland (Agyare et al. 2015a; MLNR 2012). The CREMA concept further supports climate change mitigation and carbon projects (Asare et al. 2013). Creating conservation awareness ranks high in the objectives and is reflected in appreciation of non-economic values concerning wildlife conservation among members (Robinson and Sasu 2013).

#### Adaptive management and continual learning

The CREMA is built on adaptive management principles, with monitoring and evaluation being “recognized as important aspects of its implementation and development (WD 2000, p. 10). This involves wildlife, trade and trend monitoring to guide management decisions and interventions (Ibid). Asare et al. (2013) argue that its democratic governance structure based on traditional beliefs and values enhances community consensus building, decision-making and problem solving hence adaptive management. How adaptive management materializes on the ground is however hardly documented, the exception being action learning process advocated by IUCN (Barrow et al. 2016; Baruah et al. 2016).

#### Polycentric governance

CREMA embodies a paradigm shift from conventional state-run conservation in restricted forests or wildlife reserves to inclusion of local resource users as lead managers of their natural resources. It explicitly embarks on polycentric governance (Agyare 2013) by integrating statutory (Wildlife Division, MLNR) and customary actors (traditional authorities, the Stool) and through decentralization of authority to district-level authorities and devolution of NRM to local CREMA communities. CREMA structures primarily consists of a Community Resource Management Committee (CRMC) and its Executive Committee. The CRMC comprises volunteering farmers and landholders registered to the CREMA, while the Executive Committee comprises CRMC members willing to stand elected by their peers, with roles rotating on a 3-year basis. Members are responsible for steering and overseeing the daily operations and decision-making within the CREMA (Asare et al. 2013), which includes drawing up and enforcing the constitution and by-laws with support from the Wildlife Division and District Assemblies. The latter are important in establishing CREMA constitutions as they offer legislative support and experience during the formation process (WD 2004a). Once the by-laws are recognized by the respective District Assembly, a CREMA is approved by the Wildlife Division and the Ministry of Lands and Forestry (WD 2004a, b; Nyame et al. 2012; Asare et al. 2013). The entire governance process is strongly embedded in local governance and community structures and value systems (Asare et al. 2013). CREMA communities are autonomous in their daily operations; the Wildlife Division and District Assemblies facilitate CREMA communities to set up a constitution and by-laws. The Wildlife Division oversees the CREMA operations and is responsible for renewal of the Certificate of Devolution (WD 2004b). The risk of creating new elites (mainly staff of externally funded NGOs) with limited accountability toward local interests has been documented (Baruah 2015), yet within the same cases, action learning has helped overcome such governance issues (Baruah et al. 2016).

#### Multi-stakeholder negotiation

Once power is devolved through the Certificate of Devolution, CREMA communities are autonomous in their negotiation and decision-making regarding resource use and conservation activities. Interactions and negotiations are mainly between local level actors, i.e., smallholders, land owners, the CREMA collective and district governments (Agyare et al. 2015b). When external NGOs are involved (e.g., IUCN), links are often established with other relevant actors such as the Administrator of Stool lands, or multi-stakeholder forums such as the District Forest Forum or Ghana Forest Watch (Nyame et al. 2012).

#### Stakeholder capacity building

Capacity building comes through internal objectives of the CREMA itself and may involve the assistance of external institutions, e.g., the Tourism Board and NGOs (Eshun 2010). Popular CREMA activities such as eco-tourism enhance the capacity of community members by engaging in administrative and business management ventures.

## Discussion: The Schemes’ Alignment with Design Principles for Integrated Landscape Approaches

All three NRM schemes are embedded within mosaic landscapes of continually interacting land uses, whether through agroforestry systems common throughout Burkina Faso, or a mosaic of forest and wildlife reserves with food (cassava, cocoyam, and plantain) and tree crop farming (cocoa and oil palm) in Ghana. All three landscapes are impacted by deforestation, biodiversity loss, climate change, and persistent poverty—solutions to which may be sought via integrated approaches. However, the CREMA is the only scheme explicitly embarking on multifunctionality and addressing trade-offs between conservation and development aims. This highlights the potential for the CREMA as a potential entry point for implementing integrated approaches.

As far as CAF forests are concerned, there are insufficient linkages between NRM and broader development strategies, despite these forests being historically embedded in multiple land-use mosaics of agroforestry, crop production and agro-pastoralism. Burkina Faso’s current poverty alleviation plans prioritize agricultural expansion and intensification, which can exert further pressure on forests and non-agricultural land (Foli and Rabdo 2016). Similar pressures are also present in Ghana, where agricultural policies enhance the expansion of tree crops (Ros-Tonen et al. 2015). In order to overcome such pressures the wider land uses within these mosaic landscapes need to be reconsidered as holistic interacting entities. An example of an entry point for greater integration in Burkina Faso is the clustering of land uses within the same jurisdiction as the CAF forests.

In Ghana, current pilots to integrate shade-tolerant NTFPs and food crops in the MTS and plans to further expand this toward off-reserve areas^4^ signal an important step forward in reconciling timber interests of the Ghana Forestry Commission with farmers’ need for short- and mid-term income and food security. Institutionalizing these pilots could facilitate an integrated approach toward landscape restoration.

CAF and MTS have seen changes resulting from past failures (Table 2), but again it is only the CREMA that explicitly embarks on adaptive and negotiated management led by local resource users—both in intention (WD 2000) and practice (Baruah et al. 2016). The CAF has transitioned from existing as a donor-funded intervention to a self-sustaining resource co-management scheme through revenue generated from fuelwood exploitation. However, there is no reciprocal flow of information or recognized platform where stakeholders can meet to exchange information or discuss the implications of competing land uses and landscape changes. Furthermore, there are insufficient resources allocated for periodic updating of the conservation of NRM capacity of the CAF technical unit. The MTS slowly progresses toward collaborative learning, e.g., through IUCN’s action learning approach (Barrow et al. 2016) or the TREEFARMS project led by the Resource Management Support Center of the Forestry Commission.^5^ However, participants in a workshop held in January 2017 acknowledged that ‘procedures to change the bureaucracy are slow’ (pers. obs.), meaning that adaptive management and continual learning are not yet institutionalized.

The polycentric governance paradigm has grown out of the co-existence of statutory and customary arrangements, and the influence of globalized policies and interests regarding conservation in response to climate change and unprecedented deforestation (Nagendra and Ostrom 2012; Wyborn and Bixler 2013). All schemes provide for hybrid governance arrangements to accommodate both traditional authorities and community structures and state authority. The CAF and MTS are however primarily co-management schemes where the state (the MEDD in Burkina Faso or the Ghana Forestry Commission) apply the rules of the game. CREMA communities are autonomous in their decisions once they receive their certificate of devolution. Strong links exist with other centers of decision-making such as the District Assemblies and the Wildlife Division at national level. International connections are growing with increasing donor funding and technical support from NGOs like the IUCN, while both the MTS and CREMA attracted international interest for their potential to serve as REDD pilot cases (Agidee 2011; Kalame et al. 2011; Asare et al. 2013).

All schemes are characterized by a multi-stakeholder design, but only the CREMA has institutional arrangements in place that allow for negotiated goals and change logic. The CAF and MTS are characterized by rather rigid decision-making structures in which state organizations have the biggest influence. Although CREMA’s institutional design is such that it accommodates negotiations, these are restricted to the local CREMA level. Where national and international NGOs engage in the scheme, links are established with platforms like District Forest Forums and the national-level civil society coalition Forest Watch Ghana (Nyame et al. 2012).

To participate effectively in NRM and respond to landscape dynamics, platforms are needed to exchange knowledge and experiences (Sayer et al. 2016; Ros-Tonen et al. 2015). No such platforms are in place in any of the schemes; the CAF and CREMA depend on NGOs for capacity building, and where capacity building occurs in the MTS it is restricted to seedling production and tree planting, rather than targeting improved learning capacity and negotiation skills (Opoku-Boamah and Takayoshi 2011). In the same way, assessments of the socio-economic performance of the scheme are scarce (ibid.).

**Fig. 1:**
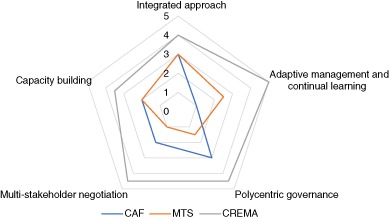
Alignment of the NRM schemes with principles for integrated landscape approaches

Based on the scoring in Table 3, Fig. 1 indicates how these schemes are currently aligned with the principles of landscape approaches. This study did not set out to use the principles as a box-ticking exercise, but to identify the strengths and weaknesses when the schemes are considered as entry points for landscape approaches.

## Conclusion

This paper explored whether, and how, three NRM initiatives in Ghana and Burkina Faso that integrate landscape and livelihood objectives of local populations can provide entry points for the implementation of integrated landscape approaches. We explored this potential by analyzing the extent to which the three schemes—the CAF in Burkina Faso, the MTS and CREMA in Ghana—align with some key design principles for integrated landscape approaches. Based on the analysis, the CREMA approach in Ghana provides the most promising entry point for implementing a landscape approach and shows how such an approach could be operationalized. However, its scale and multilevel connectedness may be too limited to guarantee effective landscape governance beyond the CREMA territory and would require up- or side-scaling, e.g., by linking several CREMAs to increase the scale of operation or by linking the CREMA to broader stakeholder coalitions (Nyame et al. 2012).

Inversely, based on their scale, the CAF and MTS represent potential integrated landscape approaches in which different and often competing land uses are interacting with one another. These interactions, as we have shown, are either through the fluid movement of actors across land uses in fulfilling livelihood objectives (CAF) or through the inherent re-design of the scheme to enable farmers to benefit from resource management efforts (the MTS).

Improving the interconnectedness of land uses in the CAF requires platforms of collective deliberation on issues of rural development, resource governance and climate change in which different land users are represented. This will reflect the reality of how rural actors utilize and preserve natural resources on the ground.

A lack of long-term funding and economic incentives threaten the durability of the MTS, while hierarchical governance arrangements impede a genuine transformation toward collaborative decision-making, power sharing and institutional diversity (Ros-Tonen et al. 2014). Only if the lessons from collaborative learning processes such as in the TREEFARMS project are internalized and rolled out, are there prospects for evolving into an adaptive and learning scheme that is open to integrated and multi-stakeholder approaches at the landscape level.

Concrete monitoring and evaluation mechanisms are key features missing from all three schemes. Monitoring contributes to identifying weaknesses within NRM schemes through evaluation of their biodiversity, economic sustainability, and social relevance (Lovell and Johnston 2009). The importance of monitoring has further relevance since all of three schemes have been established as a response to failure of past conservation strategies. A landscape approach to NRM includes a continual learning and adaptive management cycle that enables actors to respond to changes in the natural resource base (Sayer et al. 2013).

The types of knowledge deemed relevant in NRM schemes can influence the level of involvement of local user groups who often possess locally specific knowledge (Clark et al. 2016). Often, state forestry and land-use planning institutions conduct conservation programs based on universal scientific knowledge. Only the CREMA initiative in Ghana explicitly mentions the importance of local knowledge and practices for the conservation and sustainable use of natural resources. In this respect, we argue for platforms of knowledge exchange between different knowledge systems and governance levels (c.f. Ros-Tonen et al. 2015). Such platforms can act as catalyzers by providing “an arena for knowledge co-production, trust building, sense making, learning, vertical and horizontal collaboration and conflict resolution” (Berkes 2009, p. 1695). Accommodating such platforms requires organizational and institutional flexibility to provide space for informal knowledge brokering, experimental learning, and iterative approaches. This flexibility seems to be the greatest challenge, although the CREMA approach in Ghana provides glimmers of hope.

## Footnotes

1 Forest data on Burkina Faso is sparse and inconsistent as a result of different methodologies applied in forestry studies (Westholm and Kokko 2011).

2 Ghana’s Forestry Commissions consists of the Forest Services Division (FSD), the Wildlife Division (WD) and the Timber Industry Development Division (TIDD) and has the Resource Management Support Center (RMSC) as its technical wing.

3 See http://fcghana.org/treefarm/index.php/about-us, accessed 14 Jan 2017.

4 See http://fcghana.org/treefarm/ accessed 3 Feb 2017.

5 See http://fcghana.org/treefarm/ accessed 3 Feb 2017.
